# A Web-based graphical user interface for evidence-based decision making for health care allocations in rural areas

**DOI:** 10.1186/1476-072X-7-49

**Published:** 2008-09-15

**Authors:** Nadine Schuurman, Margo Leight, Myriam Berube

**Affiliations:** 1 Department of Geography, Simon Fraser University, Burnaby, Canada; 2 Computer Science, Simon Fraser University, Burnaby, Canada; 3 Department of Geography, Simon Fraser University, Burnaby, Canada

## Abstract

**Background:**

The creation of successful health policy and location of resources increasingly relies on evidence-based decision-making. The development of intuitive, accessible tools to analyse, display and disseminate spatial data potentially provides the basis for sound policy and resource allocation decisions. As health services are rationalized, the development of tools such graphical user interfaces (GUIs) is especially valuable at they assist decision makers in allocating resources such that the maximum number of people are served. GIS can used to develop GUIs that enable spatial decision making.

**Results:**

We have created a Web-based GUI (wGUI) to assist health policy makers and administrators in the Canadian province of British Columbia make well-informed decisions about the location and allocation of time-sensitive service capacities in rural regions of the province. This tool integrates datasets for existing hospitals and services, regional populations and road networks to allow users to ascertain the percentage of population in any given service catchment who are served by a specific health service, or baskets of linked services. The wGUI allows policy makers to map trauma and obstetric services against rural populations within pre-specified travel distances, illustrating service capacity by region.

**Conclusion:**

The wGUI can be used by health policy makers and administrators with little or no formal GIS training to visualize multiple health resource allocation scenarios. The GUI is poised to become a critical decision-making tool especially as evidence is increasingly required for distribution of health services.

## Background

### The move to evidence-based public policy

Policy makers are increasingly recognizing the importance of evidence-based decision making – based on integrating results from the best available systematic research with seasoned professional judgment and expertise. Evidence-based decision making (EBDM) requires a commitment of resources to support the identification of pertinent questions and the research initiatives most appropriate to answer them. For issues requiring broad-based consultation with constituents and federal-provincial partnerships, this approach ensures that a defensible assessment methodology, based on the best available evidence, is brought to bear in supporting the choice of policy or program [1-5].

The benefits to health policy of EBDM are many: greater consistency in decision making; a stronger likelihood of improved health outcomes from decisions based on reputable evidence; the potential for more cost-effective solutions with supporting evidence; program performance monitoring criteria that allow comparison with other programs; and the opportunity for further learning from experience [2,3,5-7].

### Spatial decision support systems (SDSS) as an aid to evidence-based policy making

Geographic Information Systems (GIS) offer a suite of spatial tools and methods that allow the user to analyze the spatial dimensions of health service location. GIS can be used to map sites, as well as build scenarios of different service arrangements. It is a particularly useful application for understanding the spatial dimensions of health care service delivery, and in particular the relationship between health outcomes and accessibility [8,9]. In many instances, GIS has been used to plan health care service delivery models as well as the optimal arrangement of health care facilities [10]. In order to execute these analyses, however, the ability to link health care services (hospitals) with potential patients is required.

GIS is a component of many Decision Support Systems (DSS) – which range from DSS generators to the latest flexible modelling tools based on query systems, spreadsheets, statistical software packages and GIS programs. DSS tools include powerful modelling functions; data streamlining capabilities and management features to access internal and external data; analytical capabilities and visual representations of relationships; and powerful but simple user interfaces [11,12]. Decision making problems with a large number of decision alternatives and outcomes that vary spatially require Spatial Decision Support Systems (SDSS) with large data management and integration capacity [13-16]. Geographic location can be the key feature in making data meaningful for decision makers by linking different datasets by their spatial commonalities [17]. Prior to accessible GIS tools, considering the relationship between factors influencing a decision, for example whether to close a hospital ward, was a mathematical and economic modelling exercise with limited opportunity for the decision makers to rework scenarios after the problem was defined [18].

### Making SDSS accessible in health services policy: employing a Web-based Graphical User Interface (wGUI)

A Graphical User Interface (GUI) designed to reduce cognitive load through a more structured presentation of strategic information can increase the comfort level with decision making activities. User-supportive GUI design results in a wider deliberation of options [19] through reduced data framing bias [20,21]; more successful concept communication [22,23]; and ease of user-constructed scenarios [24-26]. Decision makers explore relationships that are otherwise obscure when the decision maker is in control of the scenario generation [19,27]. The lower costs of a Web-based GUI (wGUI) supported by SDSS can increase the stakeholder involvement in health service policymaking by reducing the facilitation and planning time needed to address a specific issue [12,13,20,28].

GIS support for evidence-based health policy decision making has been constrained by reliance on GIS specialists, limiting the opportunity for considering all possible decision outcomes [1,25,29]. GIS must continue to perform traditional functions, but the training expense, time commitment, and cost of equipment with suitable software are often barriers for users [30-32]. Removing barriers to wide scale adoption of GIS as a decision making tool requires reduced training requirements, intuitive use of the software platforms and low cost access to information. If human-factor guidelines are considered, i.e. "users' platform-independent access to georegistered data, metadata, and other related information via a windows, icon, mouse...user interface" [27], the use of the Internet becomes a desirable venue.

The development of a Web-based SDSS tool is consistent with current global changes in the Web. Web 2.0 broadly refers to a new generation of Internet services and technology. This *second wave *of the World Wide Web [33] has revolutionized communication via the Internet just as the original Web revolutionized information dissemination and access. Web 2.0 promises to democratize the Internet in a way not seen before and to a large degree this is being achieved through participation amongst Web users [34,35]. The goal of Web 2.0 is a richer, more complete Web experience available to all Internet users. While there is debate over what Web 2.0 truly comprises, several key themes have emerged as the term has entered widespread use. 'The participatory Web' as opposed to 'the Web as information source' is frequently attached to descriptions of Web 2.0, of which user-created content and collaboration are the hallmarks. In fact, the idea that the average Internet user can create content is considered an essential element of Web 2.0 [36]. In comparison with the previous iteration of the Web, the new Web encourages significantly more interaction between users [37]. In addition, open access and open source are important themes for Web 2.0 [38].

The term Web 2.0 is more than just a paradigm for describing different patterns of usage; it is also a technology paradigm [39]. A key theme of Web 2.0 is the 'Internet as platform.' Cheung *et al*. [40] outline the applications that define Web 2.0: *rich Internet applications, collaboration tools, user contributed content databases, and integrative technologies*. The advent of Web-based applications promises to reduce the dominance of proprietary software manufacturers, as these tools are often free or at least provided at a significantly reduced cost compared with their traditional desktop counterparts. Given the rapid shift of the Web to a highly socialized, interactive environment, it is inevitable that SDSS will migrate to the Web as the platform of choice. The wGUI is a preliminary instantiation of the Web 2.0.

Web applications such as a wGUI can support cross-platform analysis and data sharing among decision making groups of diverse technical skills and physical locations [33]. Access to shared information is possible at any time and place with minimal infrastructure supports through client-server architecture. Web client programs (browsers) are relatively simple to use because document mark-up language ensures consistent information presentation across individual hardware and software platforms. Decision makers are accustomed to pervasive browsers and no additional training or software maintenance is required to support their use of this venue for SDSS delivery. A wGUI is an innovative means of using Web 2.0 technologies to facilitate evidence-based health policy decision making.

### A new approach to health policymaking

#### Employing a wGUI to facilitate health policymaking

The drive to more inclusive decision making in health care policy and procedural processes has challenged the expert community to develop venues for rapidly informing decision makers. Not only must information be readily available, there is a need to inform non-expert decision makers of the relevance of different information and how the data has informed past decisions. One of the means of providing distributed decision makers with differing expertise with a common framework for decision making is a wGUI. In this instance, the data retrieval, analysis and processing for the wGUI is provided by a Web-enabled GIS tool that incorporates an expert understanding of the problem and a range of solution sets. Adjectives describing a good user interface include consistent, comprehensible, natural, responsive, self-explanatory, efficient, flexible, effective, and rewarding.

Bhargava, Power et al broadly classify a wide range of customized and generic DSS into five families of DSS tools. Data driven DSS organize, analyse and retrieve large volumes of data using database queries and use online analytical processing (OLAP) techniques. Model-driven DSS represent decision models and through analysis, optimization, stochastic modeling, simulation, statistics and logic modeling, provide analysis support. Web interfaces have expanded the DSS applications to open ended problems with fuzzier choices in the form of three other types of DSS that have now become widespread. Communication driven DSS link multiple decision makers over space and time. Knowledge driven DSS assist with selection of alternatives, such as medical expert systems and scenario generators. Document driven DSS integrate a variety of storage and processing technologies to provide document retrieval and analysis {Bhargava, 2007 #299}.

In 1995 Jones [34] identified the seamless integration of user interface and DSS functionality as a key development in DSS. User interface had been a secondary consideration with the widely used data driven and model driven DSS tools. SDSS adoption research, however, challenges the idea that user satisfaction with Web-enabled DSS tools is primarily related to systems and information quality [12,23,28,35-38]. Rather, studies have shown that SDSS users experienced self-efficacy gains when working with user interface features that prioritized information presentation, resulting in perceived efficiency and decision quality improvements. The time-and-effort savings of an intuitively designed user interface mitigate a perspective that SDSS may take too much time and effort to use [24,26,39].

Once the need for a wGUI is established, it is important to ensure simple and clean design to maximize the user potential. Elvins and Jain summarize the factors of good GUI design and support the increasing use of wGUI [27]. One of the goals of wGUI for SDSS is minimizing the need for GIS technical knowledge. Well-designed user interfaces adopt a conceptual model that is familiar, and incorporate easy to understand and appropriate visual metaphors with a simple and uncluttered layout. An important feature of the wGUI is being accessible to users with varying abilities through the provision of interactive help functions. There is always the option, as the users become more adept, of turning off these functions. A well-structured user interface will minimize the amount of user work-arounds or problem solving.

An illustration of how a wGUI could address the expert concerns is found in a review of a collection of SDSS tools for integrated coastal management that involves marine, estuarine, wetland and coastal systems in topical areas. The adoption of SDSS tools the key points of success are the involvement of the end user in the development of the tools, design for end-user needs, a flexible adaptive and updatable system with an easy to use interface that requires limited time invested to learn the system{Westmacott, 2001 #311}.

Execution of tasks using SDSS – and their results – need to be reliably predictable. This is especially true when there are several ways to achieve the same results. A wGUI limits the possible ways of working with SDSS and as a consequence, reduces the number of ways it can produce incorrect results. User errors will still occur but it is easier to address these when they do if there are limitations on what modifications to the SDSS and data sources are possible.

### Optimal location and ensuring maximum access to health services: creating a wGUI to facilitate health services location

In response to the specific needs of health policy within British Columbia, we have developed a wGUI designed to assist BC health policy makers in making better informed decisions about service reallocations and hospital closures or openings affecting rural regions in BC. Potential users of this wGUI include *British Columbia Ministry of Health *Medical Officers and Information Officers who are charged with making decisions about service provision. A number of allocation decisions are devolved to the level of the regional Health Authority; thus the five Health Authorities in the province are also likely users.

The wGUI is designed to provide information about the geographical extent of service catchments around health services in rural and remote British Columbia (e.g. the entire province excluding the two metropolitan areas of Victoria and Vancouver). A catchment is a designated geographical area around a service. In addition, the wGUI provides information about the total number of people in each existing or hypothetical service catchment as well as percentages of the population *not served *within the specified road travel time.

In this case, the catchment sizes were defined as one, two and four hour drive times. These designations reflect the standards set by the *British Columbia Ministry of Health *specifying the maximum acceptable distances to any given health service. The provincial standards specify, for instance, that access to emergency services (24/7/52) should be provided within a one hour travel time for 98% of the population [48]. Likewise, access to basic inpatient hospital services should be within two hours travel time for 98% of the population [48]. Finally, the province's standards of accessibility for services specify that core specialty services should be available within four hours travel time for 98% of residents [48]. There is evidence that time to definitive care is an important factor in outcome. In the case of trauma, for example, research has indicated that access to service within the "golden hour" has a profound positive effect on outcome [49,50].

Using the wGUI, planners can determine the extent to which the province is fulfilling its own mandate for service provision to 98% of the population within specified travel times for specific services. The wGUI can also be used to support decisions about hospital closures, openings and service reallocations in rural and remote regions in BC – as it provides numbers of people served for a given health service within specified times. The wGUI is designed to allow health planners and administrators to determine the optimal location of health services based primarily on the criterion of serving the largest possible population within certain road travel time catchments.

The initial version of the wGUI illustrated here allows catchment calculation and associated population reporting for regional and provincial levels of trauma and maternity services throughout the province of British Columbia. In addition, it allows health policy planners to analyse scenarios for *all *hospitals in British Columbia – without specific service differentiation.

At present, the wGUI incorporates detailed service data for trauma and maternity services – though more service baskets are currently being added. In addition, it excludes the two Central Metropolitan Areas (CMAs) of Vancouver and Victoria as there is considerable overlap between services in urban areas so access in these regions is a less pressing issue.

## Results

The wGUI we have developed was created to enable health policy makers and administrators in BC to generate evidence to support their decision making process, specifically about the location and allocation of time-sensitive service capacities in rural regions of the province. It was designed to facilitate use by a broad group of stakeholders with varying levels of expertise across a range of locations.

**Figure 1:**
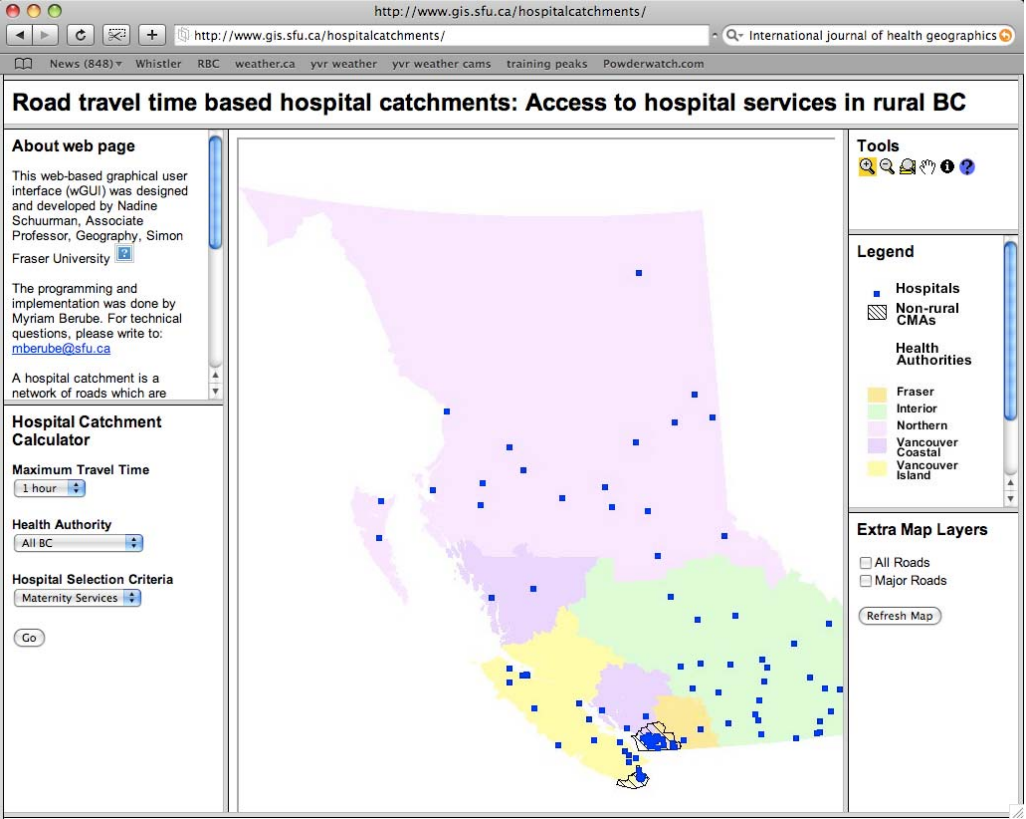
The wGUI opening window.

When the wGUI is launched, the opening screen reveals seven screens. The lower left screen illustrated in Figure 1 allows the user to choose a maximum travel time, a Health Authority and a set of hospitals. The Maximum Travel Time options are one hour, two hours or four hours while the Health Authority options comprise BC's provincial health regions: Fraser, Interior, Northern, Vancouver Coastal, Vancouver Island and "All BC". The central screen in the main window contains a map of British Columbia divided into the five Health Authorities (i.e. the chief administrative planning bodies), showing all the roads and hospitals in the province (Figure 1). Users may select a set of BC hospitals, by name or services offered, and determine the number of people in a particular Health Authority (or all of BC) within a specified travel time of each hospital. The resultant analysis will illustrate the number of people served and conversely those excluded from services within the travel time defined for the scenario. Users may also run analyses with hospitals added or removed from the initial selection – in order to visualize effects of health services closures or openings. They can then compare the populations served within hospital catchments before and after each change.

**Figure 2:**
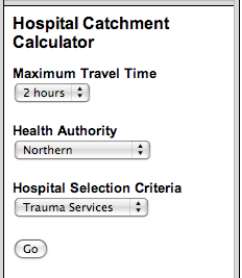
Choosing trauma service catchment parameters.

**Figure 3:**
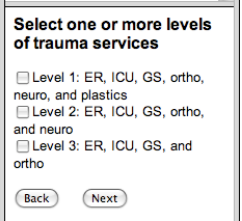
Choosing trauma service levels.

The subsequent wGUI window depends on the user's hospital selection criterion. For example, if the user selected a two hour maximum travel time for trauma services in the Northern Health Authority (as illustrated in Figure 2), then a new window illustrated in Figure 3 would open allowing the user to refine the basket of trauma services selected. The trauma services choices are consistent with level one (tertiary), level two (regional) and level three (basic) services.

**Figure 4:**
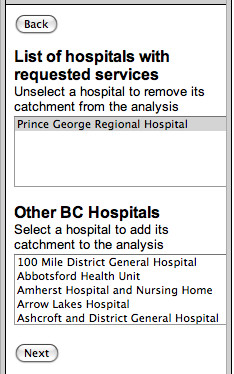
**The list of hospitals with the selected trauma service level – as well as other BC hospitals.** The latter list can be used to select additional hospitals where policy makers may want to model added services to determine the effect on numbers of people served within the selected two hour travel time catchment.

Alternatively, if maternity services were chosen, a window with options for levels of maternity service from midwife to full obstetrical services follows. In each case, the user is presented with a list of hospitals that fit that service criteria as well as a second list that enables them to model alternate scenarios in which hospital services are added. The user can choose to delete a hospital from the top list of those that fulfil the service criteria to determine what percentage of the population would be adversely affected by service removal. These choices are illustrated in Figure 4.

**Figure 5:**
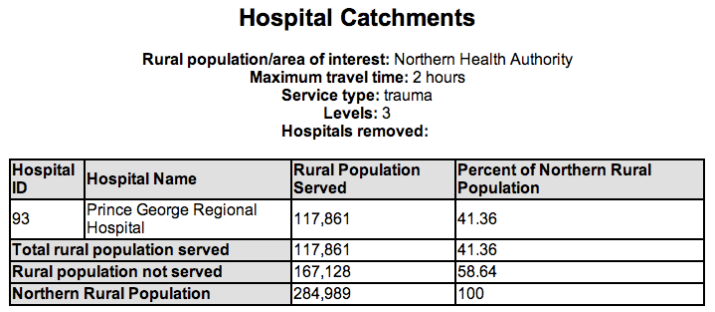
**The table generated with the "View Catchment Report" button is pressed.** This table illustrates the total population served within the two hour catchment travel time for the selected service in the selected Health Authority. In this case, 41.36% of the population of the extensive Northern Health Authority is *not served *within two hours travel time of the most basic level 3 trauma services. This report communicates important information about the vulnerability of rural and remote populations and serves to inform administrators of where health services may be needed.

When "Next" is selected in the Figure 4 window, a "View Catchment Report" button is presented. When that button is clicked, an html page opens in the user's Web browser with a table displaying the hospitals which satisfy the user's choices. The resulting table from this sample catchment calculation is illustrated in Figure 5.

**Figure 6:**
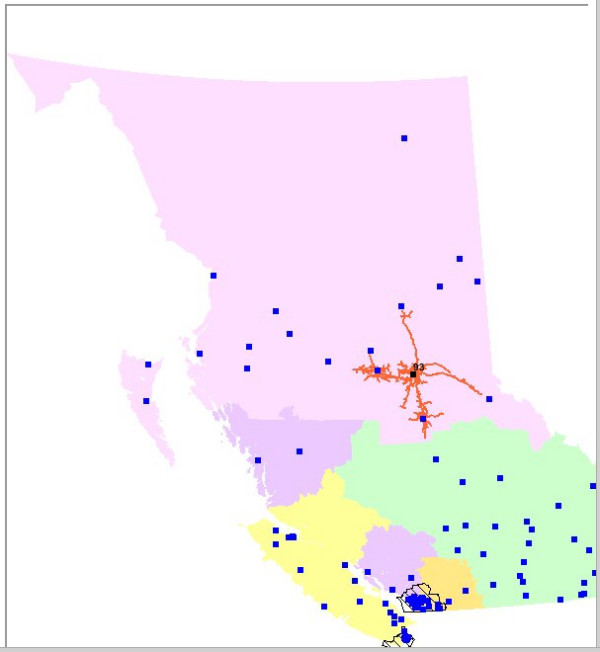
**The catchment map generated by the sample query. **In the case the catchment – shown in orange – represents a two hour road travel time from Prince George Regional Hospital in British Columbia's Northern Health Authority (NHA). Prince George is the only hospital to offer any trauma services in the NHA.

In addition, a map of the catchment around the selected hospital(s) is drawn in the central wGUI screen – as illustrated in Figure 6.

**Figure 7:**
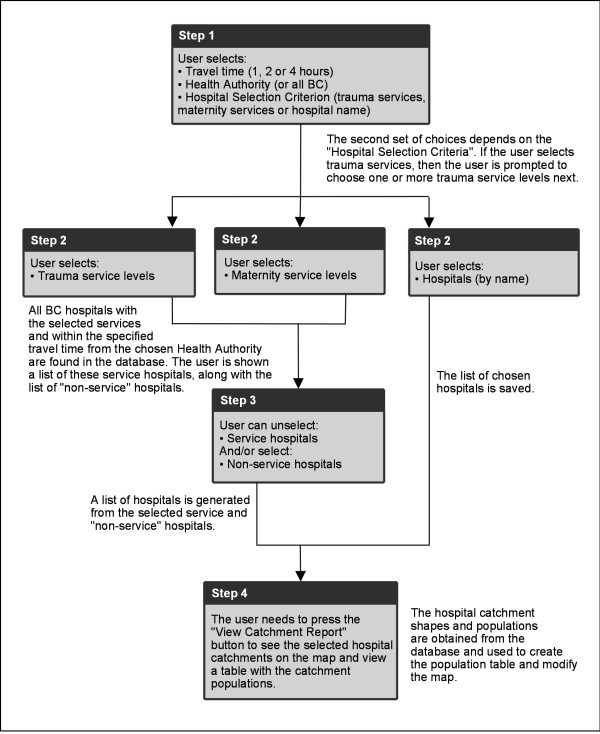
**The wGUI presents the user with a series of choices in order to direct their query.** The user steps are summarized in this flowchart and underlying processing is explained for each step.

The wGUI allows the user to select British Columbia hospitals by name or services offered, as well as by travel time from populations anywhere in British Columbia or within a specific Health Authority. Moreover, users can modify the list of suitable hospitals by removing or adding hospitals to simulate the closure or addition of services. The wGUI creates a table with the final list of hospitals and the populations within the chosen travel time and health authority. The table also lists the populations within the chosen Health Authority (or all BC) that are outside the hospital catchments. If there are populations that live within the chosen travel time of more than one suitable hospital, they are assigned to the closest one. The wGUI adds graphical representations of the hospital catchments to a map of British Columbia. The user can zoom in, zoom out and pan the map, as well as get hospital information by clicking on a hospital square. Figure 7 illustrates the application flow and describes the underlying algorithmic processes.

The wGUI is built on a foundational ArcMap GUI. The latter refreshes the map image more quickly and allows the user to perform further analysis on the hospital catchment features by using the many ArcMap tools and by adding other data to the map. However, it also requires an expensive ArcGIS Desktop license and some knowledge of GIS software. The functionality of the wGUI may be more limited but anyone with a password and a Web browser can use it and additional functionality will be added in the future as the GUI is developed.

## Discussion

There are a number of benefits to developing a wGUI for policy makers and health administrators to use in allocating resources in rural and remote areas where catchments seldom overlap – especially given their constrained financial resources. The wGUI is readily accessible from the Web, requires no expertise in spatial analysis, and is easy to use. It delivers clear results in map form as well as providing the population figures needed to support decisions about allocation that will benefit the greatest numbers of people. In a policy environment that is increasingly espousing the value of evidence-based decision making, such tools are especially important.

In the Canadian province of British Columbia where mountainous regions constrain car travel, incorporating travel time calculations into the wGUI to determine level of access imbues the results with greater reliability. This is especially pertinent as fewer than 7% of traumatic injuries, for example, are airlifted from rural and remote areas. We expect that the wGUI will prove useful in other rural and remote jurisdictions nationally and generally provide a critical addition to support evidence-based decision making for health care.

Decision making tools need to be designed so they do not integrate hidden errors and thereby facilitate faulty interpretation [51]. There is a risk that a Web-based SDSS tool might allow users lacking in GIS skills to draw the wrong conclusions, leading to non-rational decisions. Ideally, a user should only be able to produce valid results and maps. In this case, the wGUI is not designed to operate as a decision making framework and is therefore not providing information subject to interpretation. It serves as a check against population access to health services within mandated road travel time, returning information on the spatial extent of one, two and four hour road travel time catchments. In addition, it provides supporting tables that illustrate the percentages of total population served and not served within the query region. The nature of information rendered, therefore, does not allow for misinterpretation. Should wGUIs based on GIS become more prevalent, it will be important to ensure that they are designed to prevent users without broad spatial awareness from reaching the wrong conclusions.

### Limitations

Perhaps the most obvious limitation to the wGUI is the fact that many health allocation decisions are made based on a large number of internal and political pressures that are unrelated to evidence. Many decisions are made independent of rational evidence with respect to resource allocation. Indeed, there is evidence that models are sometimes used only to buttress decisions – once they are already made [52]. Perhaps the greatest danger is not lack of evidence-based policy but the generation of policy-based evidence [52].

Additional limitations include reliance on Statistics Canada population data which, although quite accurate, is collected only every five years. Also, at present, only two specific health services (trauma and maternity) are included in the model though our group intends to add cardiac, ER, palliative and oncology services shortly.

## Conclusion

The wGUI described in this paper was constructed specifically to aid hospital administrators and health policy makers in making decisions about allocation of resources. The wGUI is accessible to users with little or no GIS experience and is easy to use over the Web. It permits users to visualize the geographical area served by a specific hospital service such as trauma or rural maternity. In addition, it provides accurate population estimates of people served within a one, two or four hour travel time from a specific hospital service. Finally, the wGUI enables policy makers to create scenarios in which a hospital is added or removed – and visualize the resulting impact on the population served. This tool is poised to become an integral component of evidence-based decision making for the allocation of health services when the goal is to serve the maximum number of people in dispersed rural and remote regions.

## Methods

### Data

Five datasets were used to develop the GUI: BC hospital data, BC road network data, BC census block population data, BC Health Authority data and BC Census Metropolitan Area data. The hospital data included attributes indicating the presence or absence of various health services (e.g. neurosurgery; ICU). The road features were associated with vehicle travel times for both directions. The census block centroid point features linked to population values were used to determine populations within specific travel times of a hospital. BC Health Authority polygons allow the option of permitting the user to restrict analysis to particular Health Authorities. Finally, the BC Census Metropolitan Areas (CMAs) were used to restrict the analysis to rural areas of BC, by masking the Vancouver and Victoria CMAs.

Five new datasets were derived from the five datasets listed above: an origin-destination cost table, a Health Authority population table and 3 hospital catchment feature classes for one, two and four hour travel times.

The data used by the ArcMap GUI was stored in an Access geodatabase. When the wGUI was created, this data was copied to an Oracle database to allow concurrent multi-user access. An ArcSDE Server was used as the gateway between the GIS software and the Oracle database.

### GUI construction

#### Creating the road network dataset

ESRI's ArcCatalog 9.1 and Network Analyst tools were used to create a geodatabase road network dataset. Before building this network dataset, attributes were added for travel time in each direction along a road segment. To calculate these times, the segment length, speed limit and presence of a stop sign or light were considered. Stop signs were assumed to add 30 seconds to the travel time while lights were assumed to add one minute. Some road features were also removed prior to network dataset creation. Road features with a road classification of lane, ferry or trail were removed. Roads in the non-rural Central Metropolitan Areas (CMAs) that provide the fastest access to each non-rural hospital from rural areas were identified. All other roads in the non-rural regions were removed to decrease the density of the road network in this region and thus speed up network analysis involving non-rural hospitals.

#### Creating the origin-destination cost table

ESRI's Network Analyst New OD Cost Matrix tool (in ArcMap 9.1) was used to create the origin-destination cost table. The BC hospital points were loaded as destinations and the rural BC census block centroid points within 2.5 kilometres of a road were loaded as origins. Travel times less than 4 hours were calculated for all hospital and centroid (within 2.5 kilometres) combinations. Fields for census block population, census block health authority, hospital maternity service level and hospital trauma service level were added to the table created by the New OD Cost Matrix tool.

#### Creating the hospital catchment feature classes

Hospital catchments, or service areas, were created in ArcMap 9.1 using the Network Analyst New Service Area tool. This process required hospital point features as well as a road network dataset. For each maximum travel time (one, two and four hours), a feature class composed of road line networks around each hospital was generated. Hospital catchments that were distributed across more than one health authority were split along the health authority boundaries. Thus, the wGUI can select catchments by hospital and health authority.

#### Creating the ArcMap GUI

Visual Basic for Applications, or VBA, is an embedded programming environment that can be used to automate, customize and extend ESRI applications such as ArcMap. Before developing the wGUI, we decided to build the GUI as an ArcMap macro using ScenarioBuilder, in order to develop and test the data and code required for the GUI. ScenarioBuilder allowed the creation of various pop-up windows that permit the user to select hospital catchment analysis options. It also adds catchment features to an ArcMap document map already showing BC hospitals, health authorities and roads.

### Porting the GUI to the Web

The ArcMap version of the GUI (described above) can only be utilized by users with ArcGIS Desktop (software which includes the ArcMap application) installed on their personal computer. The data required for the hospital catchment maps and calculations must also be located on the user's computer, clearly limiting functionality for the vast majority of potential users.

To create a Web GUI with an interactive map of British Columbia's hospitals and roads, a software application for publishing interactive maps on the Internet was required. ESRI's ArcIMS 9.1 software was used to perform this task. Furthermore, a Web server was needed to serve the Web site; a coding language for developing the Web application had to be selected; and the data needed to be stored in a database which allows concurrent access to one data layer by multiple users.

The ArcIMS software was installed on a machine running an Apache HTTP Server. Since many of the ArcIMS components were built with Java, a Java Development Kit (JDK) and a servlet engine (Apache Tomcat) also needed to be installed.

It is possible to create a Website using an ArcIMS template but this requires using javascript and ArcXML to add functionality. Since creating the Hospital Catchment Web GUI involved coding a great deal of custom functionality, it was decided to create a custom Web application using Java Server Pages. Thus, the Java Connector, which contains the ArcIMS Java Connector API JavaBeans, was installed with ArcIMS. These JavaBeans and their methods can be used in Java code to implement map related functions. The ArcSDE Java API JavaBeans were also used to query the tables in the Oracle database. The ArcSDE Java API was obtained from the ArcSDE Client software. The data were copied to an Oracle database which allows concurrent multi-user access and an ArcSDE server functioned as the gateway between the database and the GIS software.

To create the hospital and population table reports (illustrated in Figures 5, and 6), ActiveX Data Objects (ADO) and SQL were used in the VBA code to query the origin-destination cost table. The query picks all the records in the origin-destination cost table with the hospital names, maternity service levels, trauma service levels, health authority, and travel time chosen by the user. If a census block occurs more than once in all the records that satisfy the user's selections, only the record with the lowest travel time is chosen (note that more than one hospital can be less than the maximum travel time from a census block centroid). For each hospital, the census block populations are summed. The total rural population of the chosen Health Authority is taken from the HAPopulations table. If the user is interested in all rural BC populations, the population values in the HAPopulations table are summed. ArcObjects is used in the VBA code to select the catchments from the travel time appropriate Hospital Catchments feature class (one, two or four hours).

## Competing interests

The authors declare that they have no competing interests.

## Authors' contributions

NS conceptualized the study, designed the methodology, secured the data, supervised the GUI and wGUI development and wrote and revised this paper. ML researched other GUI work and spatial decision support systems. MB prepared the data, developed and implemented the ArcGIS and Web GUIs. In addition, she tested the prototypes. All authors read and approved the final manuscript.
